# Principles for developing animal models of military PTSD

**DOI:** 10.3402/ejpt.v5.23825

**Published:** 2014-08-14

**Authors:** Nikolaos P. Daskalakis, Rachel Yehuda

**Affiliations:** 1 Department of Psychiatry, Icahn School of Medicine at Mount Sinai, New York, USA; 2 Mental Health Care Center, James J. Peters Veterans Affairs Medical Center, Bronx, USA

**Keywords:** Animal, posttraumatic stress disorder, military, combat, biomarkers

## Abstract

The extent to which animal studies can be relevant to military posttraumatic stress disorder (PTSD) continues to be a matter of discussion. Some features of the clinical syndrome are more easily modeled than others. In the animal literature, a great deal of attention is focused on modeling the characteristics of military exposures and their impact on measurable behaviors and biological parameters. There are many issues to consider regarding the ecological validity of predator, social defeat or immobilization stress to combat-related experience. In contrast, less attention has been paid to individual variation following these exposures. Such variation is critical to understand how individual differences in the response to military trauma exposure may result to PTSD or resilience. It is important to consider potential differences in biological findings when comparing extremely exposed to non-exposed animals, versus those that result from examining individual differences. Animal models of military PTSD are also critical in advancing efforts in clinical treatment. In an ideal translational approach to study deployment related outcomes, information from humans and animals, blood and brain, should be carefully considered in tandem, possibly even computed simultaneously, to identify molecules, pathways and networks that are likely to be the key drivers of military PTSD symptoms. With the use novel biological methodologies (e.g., optogenetics) in the animal models, critical genes and pathways can be tuned up or down (rather than over-expressed or ablated completely) in discrete brain regions. Such techniques together with pre-and post-deployment human imaging will accelerate the identification of novel pharmacological and non-pharmacological intervention strategies.

Prior to exposure to trauma, soldiers who will participate in a combat theater are selected according to fitness criteria and undergo training, which is designed to prepare them. Nonetheless, the probability of posttraumatic stress disorder (PTSD) after combat exposure is high (approximately 40%); higher than after a natural disaster (approximately 4%) but lower than after rape (approximately 65%) (Kessler, Sonnega, Bromet, Hughes, & Nelson, 1995; Nemeroff et al., 2006; Harvey & Yehuda, 1999). PTSD evolving in combat settings frequently involves prolonged periods of hypervigilance as well as multiple incidents of trauma exposure, which contrast to a single brief and unique incident of high intensity such as a rape or a natural disaster. Military PTSD differs from civilian PTSD also in compliance or response to treatment, nature and severity of premorbid and comorbid conditions and natural history of the disease (Bradley, Greene, Russ, Dutra, & Westen, 2005; Hoge et al., 2004). Presumably biomarkers and drug-targets of military PTSD might differ from civilian PTSD (Yehuda, Neylan, Flory, & McFarlane, 2013).

PTSD research has highlighted differences related to PTSD prevalence, rather than on clinical and biological differences associated with the nature of the trauma. A large and growing literature has addressed the use of animal models to study PTSD and related syndromes with much attention to the nature of exposure and especially to the type of threats that are conceptually similar to those experienced in deployment (Daskalakis, Yehuda, & Diamond, 2013; Ursano et al., 2010). The existing models reflect aspects of single/repeated/chronic stress, escapable/inescapable stress, predictable/unpredictable stress, predator stress, social defeat stress, and fear conditioning/extinction for construct validity and a wide range of behavioral outcomes (Table 1) associated with deployment-conditions and responses (such as social withdrawal, fear and anxiety-like behavior, and anhedonia) for face validity (Daskalakis, Yehuda, et al., 2013). Yet, animal models cannot capture the totality and complexity associated with combat-related deployment; factors such as horror, disgust, repulsion, shame, guilt, feeling of responsibility, cognitive appraisal, moral/ethical attributes cannot be modeled in animals.

**Table 1:** Lists of behavioral outcomes associated with deployment conditions and responses

Behavioral domain	Example of behavioral paradigm
Addiction liability	Place conditioning
Aggression	Territorial behavior, urine marking
Anhedonia	Preference for sucrose–fat, sexual activity
Anxiety	Open field, elevated plus maze
Avoidance of trauma cues	Cat odor, auditory cues
Fear extinction deficits	Fear conditioning and extinction
Food intake	Weight, food consumption
Hyperarousal	Acoustic startle response
Memory deficits	Spatial memory
Social avoidance	Interaction test, partition test, social preference
Sustained fear	Cue and contextual fear

The majority of animal studies investigate primarily differences associated with the exposures *per se* but not individual differences in the behavioral response (Fig. 1). The source of the latter might be *a priori* genetic, sex-related, epigenetic and prior-experience (especially developmental) dependent differences (Cahill, 2006; Claessens et al., 2011; Cohen & Yehuda, 2011; Daskalakis, Bagot, Parker, Vinkers, & de Kloet, 2013; Hinton & Lewis-Fernandez, 2011; Meaney, 2001; Yehuda et al., 2010; Yehuda et al., 2014; Yehuda, Koenen, Galea, & Flory, 2011; Zoladz & Diamond, 2013; Zovkic, Meadows, Kaas, & Sweatt, 2013) or differences the stressor induces. Animal models that focus on the biological basis of individual differences in risk for PTSD following trauma exposure are particularly relevant for the military where stress exposure is expected and predicting who is at risk is potentially highly beneficial.

**Fig. 1:**
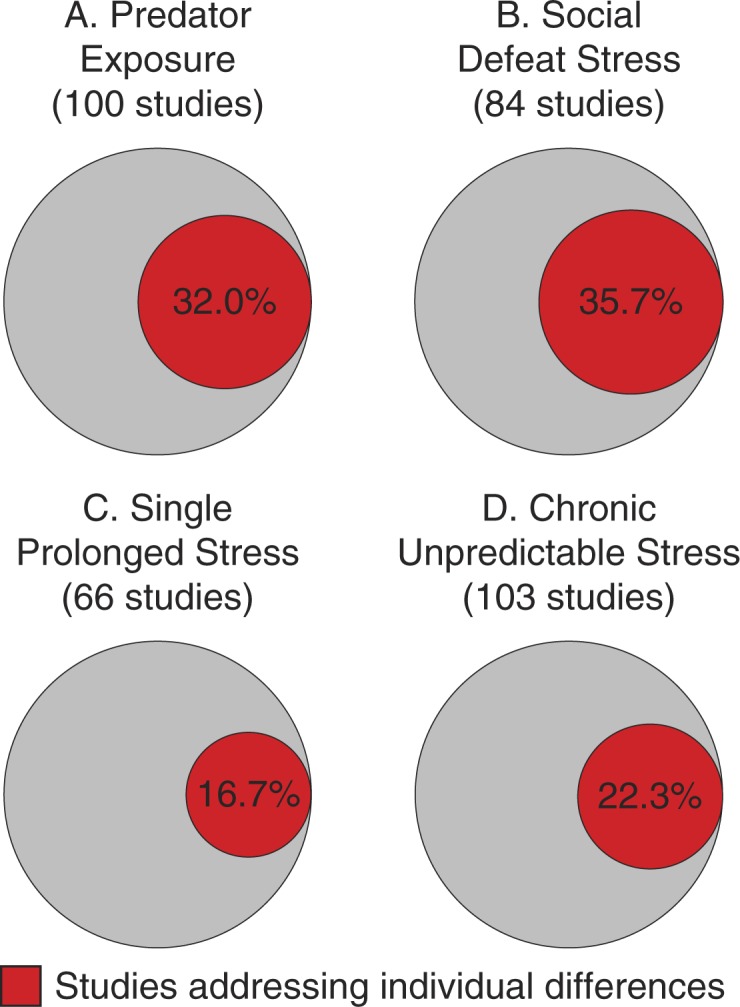
Proportional diagrams of the number of studies from a PubMed (http://www.ncbi.nlm.nih.gov/pubmed/) literature search (all references until December 2013) for: (A) (stress disorder or depressive disorder or anxiety disorder) AND animal AND predator; (B) (stress disorder or depressive disorder or anxiety disorder) AND animal AND social defeat; (C) (stress disorder or depressive disorder or anxiety disorder) AND animal AND single prolonged stress; (D) (stress disorder or depressive disorder or anxiety disorder) AND animal AND chronic unpredictable stress. The review articles were filtered out. The remaining studies were divided into studies not addressing individual differences or studies addressing individual differences (genetic, sex-related, epigenetic, or related to prior experiences).

In this paper, we will discuss various models for PTSD (Table 2) and their relevance for military PTSD.

**Table 2:** Exposure characteristics of the reviewed animal models of PTSD

Animal model	Exposure	Reference
Predator stress	1 day: 10-min inescapable exposure to a cat	Adamec and Shallow, 1993
Predator-scent stress	1 day: 10-min inescapable exposure to cat litter	Cohen et al., 2003
Predator-based psychosocial stress	31 days: - Days 1/11: inescapable 1 h immobilization & exposure to a novel cat - Daily unstable housing conditions	Zoladz et al., 2008
Social defeat stress (resident-intruder paradigm)	5–10 days: - Daily inescapable contact with a novel aggressive—resident for 5–10 min (social defeat). - Subsequent housing with sensory (not physical) contact with the intruder for the remainder of the day.	Golden et al., 2011; Koolhaas et al., 2013
Witnessed social defeat stress	5–10 days: - Daily inescapable sensory (not physical) contact with the social defeat of a novel intruder by a novel resident for 5–10 min. - Subsequent housing with sensory (not physical) contact with the intruder for the remainder of the day	Warren et al., 2013
Cage-within-cage resident–intruder paradigm	5–10 days: - Daily inescapable sensory (not physical) contact with a novel resident for 6 h - One to three random times within the 6-h sessions, physical contact with the resident for 1 min.	Hammamieh et al., 2012
Fear conditioning	1–2 days: Daily trials of unconditioned stimulus (e.g., foot-shock) paired with conditioned stimulus (e.g., tone)	Kim & Fanselow, 1992; Phillips & LeDoux, 1992
Immobilization stress and fear conditioning	7 days: 2-h immobilization stress followed by fear conditioning trials in a novel room 6 days after	Andero et al., 2011

## Predator stress

A well-validated model of PTSD, originally developed by Adamec and colleagues, involves using *predator stress* to produce enduring anxiety-like responses in rodents (Adamec & Shallow, 1993). The stressor is a supervised but otherwise unprotected exposure of a rat or mouse to a cat (10 min), which produces enduring changes in anxiety-like behavior and arousal, avoidance of trauma-related cues and social withdrawal (Adamec & Shallow, 1993). The predator stress has ecological validity as a robust and innate stressful threat for rodents, in keeping with the types of trauma that typically elicit PTSD, in that predator exposure involves actual or threatened death or serious injury. The duration of anxiogenic effects after predator exposure, as a ratio of the lifespan, is comparable to the duration of psychopathology required for a diagnosis of chronic PTSD in humans (Adamec & Shallow, 1993). Amygdala, prefrontal cortical and hippocampal circuits are implicated in the behavioral changes produced by predator stress (Adamec, Blundell, & Burton, 2005).

Variations of this model include: 1) the *predator-scent stress* model where the predator exposure is reduced to an inescapable exposure to cat litter (Cohen, Kozlovsky, Alona, Matar, & Joseph, 2012; Cohen & Zohar, 2004; Cohen, Zohar, & Matar, 2003); 2) the *predator-based psychosocial stress* model where the cat exposure is combined with immobility stress and daily social instability (Zoladz, Conrad, Fleshner, & Diamond, 2008). In work by Cohen et al., a subset of stress exposed Sprague Dawley rats develop PTSD-like phenotype, approximately 25%, and another subset is minimally affected (Cohen & Zohar, 2004). The prevalence of these post-stress phenotypes is similar in females (Cohen and Yehuda, 2011). In experiments by Zoladz et al., all exposed Sprague Dawley rats exhibit PTSD-like effects (e.g., Zoladx). This suggests a relationship between trauma characteristics and individual variation in trauma-related phenotypes.

This model has been proven to be sensitive to manipulations of single genes (e.g., CRH receptor 1; Kozlovsky, Zohar, Kaplan, & Cohen, 2012), pharmacological treatments (e.g., tianeptine; Zoladz, Fleshner, & Diamond, 2013) and secondary prevention strategies (e.g., hydrocortisone; Zohar et al., 2011). Furthermore, similar enduring changes in startle magnitude and habituation are seen in both predator-exposed rodents and humans with PTSD, offering a measure to test the cross-species translatability of these findings to patients in our human samples (Bakshi, Alsene, Roseboom, & Connors, 2012).

Regarding the applicability of this model to the nature of combat-related PTSD, predator stress clearly would be relevant to asymmetric warfare, where a soldier or combatant can be severely out-numbered and with limited reources. Additionally, every soldier or combatant, outnumbered or not, may experience an element of this type of stress in relation to the fear of the enemy.

## Social defeat stress

In the typical rodent *social defeat stress* (resident–intruder) paradigm (Golden, Covington, Berton, & Russo, 2011), a test male rodent (“intruder”) is placed in the home cage of an aggressive rodent (“resident”) for 5–10 min during which time physical fighting occurs (Koolhaas et al., 2013). The animals are then separated by a screen, which prevents further physical contact but allows all sensory stimulation to continue for the rest of the day. This process is repeated daily for several days. This type of social stress for 10 days in male rodents induces a behavioral syndrome characterized by: social avoidance; anhedonia-like symptoms (inability to experience pleasure from natural rewards) including reduced preference for sucrose, high fat diet, and sexual behavior; anxiety-like symptoms; disrupted circadian rhythms; increased addiction liability; and a metabolic syndrome characterized by increased eating and weight gain and insulin and leptin resistance. Many of these symptoms are long-lived (some persist at least 6 months after chronic social defeat stress) and can be partly reversed in some individuals by chronic, not acute, administration of standard antidepressant, but not anxiolytic, medications (Berton et al., 2006). PTSD and its co-morbidities are often consequent to repeated aggravated “social” assaults (e.g., combat) and manifest socially over time, suggesting the relevance of this repeated aggressor-exposure model to clinical aspects of PTSD. The social defeat paradigm is interesting in the context of modern warfare (e.g., Afghanistan, Iraq) where, in the absence of clear battle lines, troops are stationed within the territory of the enemy.

Importantly, 30–40% of mice (C57 BL/6 strain) sub jected to chronic social defeat stress escape most of the above symptoms. These mice are referred to as “unsusceptible” compared to the majority that is referred to as “susceptible” (Golden, Covington, Berton, & Russo, 2011). With the identification of the two subpopulations, this model was proven to be uniquely powerful to understand both: 1) the mechanisms by which chronic social stress causes symptoms in some mice, and 2) the mechanisms that explain why other mice escape these deleterious adaptations. Studies in several stress-regulatory brain regions, incl. the amygdala, hippocampus, medial prefrontal cortex (mPFC), nucleus accumbens (NAc), and ventral tegmental area (VTA), identified genome-wide changes in gene expression and chromatin structure alterations associated with susceptibility vs. resilience to chronic social defeat stress (Russo, Murrough, Han, Charney, & Nestler, 2012). For example, brain-derived neurotrophic factor and several of its downstream signaling proteins in the limbic brain were found to play a crucial role in reducing stress vulnerability (Berton et al., 2006). The WNT-signaling pathway was also involved in mediating resilience and antidepressant-like responses in the NAc (Wilkinson et al., 2011).

Witnessing death and suffering and human injury is also a relevant issue and a recent study using this model has actually highlighted this aspect (Warren et al., 2013) by developing a variant of the original paradigm, (i.e., *witnessed social defeat stress*). Here, the test mouse witnesses another test mouse of the same strain undergoing physical defeat, and then spends the rest of the day with that aggressor behind the same screen. Ten days of this witnessed stress results in nearly as robust behavioral sequelae as physical defeat, with roughly the same fraction of animals showing susceptibility vs. resilience. Such witnessed defeats seem particularly relevant to combat-related PTSD, and analogous to battlefield exposures, wherein many troops suffer severe psychological syndromes by witnessing death or maiming of their colleagues without sustaining injury themselves. Actually, exposures to human atrocities tend to even have greater impact than self-exposures (Yehuda, Southwick, & Giller, 1992).

Hammamieh and colleagues have also developed another variant of the social defeat stress paradigm. This model involves repeated exposures to a trained aggressor mouse to simulate aspects of PTSD (2012). The *cage-within-cage resident–intruder* paradigm, male mice (also C57 BL/6) are in sensory contact with trained aggressors for 6 h daily session that include one to three (unpredictable) daily physical contact periods for 5 or 10 days. During aggressor exposure, mice display less territorial behavior, increased weight, and increased body temperature. One day after the last aggressor exposure, inflammatory cardiac histopathologies are present; after 10 days, mild myocardial degeneration with fibrosis or fibroplasias are evident, while control mice show almost no cardiac abnormalities at any time. After 4 weeks, the medial frontal cortex of control mice show increased dendritic spine density, but aggressor-exposed mice showed no increase. For up to 6 weeks after the last aggressor exposure, subjects display prolonged grooming, freezing, retarded locomotion and no tail rattling, the traits of fear response to contexual cues. Activated gene modules (across blood and various brain regions) were identified in these social defeated mice (Yang et al., 2013), which taken together imply a disruption of essential cellular functions (Zhang & Horvath, 2005).

It is critical to determine sex differences in critical pathways and phenotypes related to deployment and deployment-related responses. Social stress models with females are challenged by the virtual rarity of male-typical aggression among female rodents (Albert, Jonik, & Walsh, 1992). This could be addressed by examining ovariectomized female mice from an aggressive mouse strain (e.g., SJL) primed by male steroids and trained againt non-combating females. Finally, it would be very relevant to test the impact of social hierarchy on the individual variation in the stress response to social defeat (Blanchard, Flannelly, & Blanchard, 1988).

## Fear conditioning and immobilization stress

Fear learning or extinction of fear, are certainly relevant to PTSD-like outcomes. PTSD is a condition in which processes of fear modulation are dysregulated—with enhanced fear learning, decreased safety learning and discrimination, and decreased extinction of fear (Pitman et al., 2012). Gene expression studies in a number of fear conditioning models has identified several pathways and molecular mechanisms important to fear consolidation and extinction (Daskalakis, Yehuda, et al., 2013).

Ressler and colleagues have recently used fear conditioning in conjuction with prior immobilization stress to a wooden board (IMO), to model PTSD-like behavior in mice (Andero et al., 2011) and to study resultant differential gene expression in the limbic brain (Andero et al., 2013). IMO results in a PTSD-like impaired fear extinction over a long period of time. IMO mice are unable to discriminate between safety signals and danger signals (Andero et al., 2013), analogous to PTSD patients (Pitman et al., 2012). IMO also elicits long-term impairements in spatial memory and enhanced anxiety (Andero et al., 2013). This model maybe also useful in identifying molecular changes during the immediate posttraumatic period, where there is an incubation prior to the full manifestation of behavioral symptoms (Andero et al., 2013). This is particularly relevant to PTSD trajectory since deployment-related PTSD outcomes may not be observed immediately.

The authors identified gene probes that were differentially expressed in the amygdala as a function of prior immobilization (IMO) history, and not fear conditioning *per se*. Of the identified probes, Oprl1 gene (encoding the nociceptin receptor) is highly expressed in central amygdala (CeA) whereas its expression in other brain regions is relatively low. Oprl1 mRNA was significantly up-regulated in the IMO-exposed animals compared to the control-fear condition, compared to prior IMO exposed animals demonstrating an amygdala-specific dysregulation of Oprl1 gene expression after fear conditioning and extinction in IMO mice. Finally, OPRL1 agonists, administered in CeA, could inhibit fear consolidation in mice and a DNA polymorphism within the human OPRL1 gene was associated with PTSD, altered fear learning and amygdala-insula co-activation (Andero et al., 2013).

Fear conditioning and immobilization stress could represent quite different dimensions of combat-related experience. On the one hand, soldiers will often argue that the inability to act, for example when being bombarded is far more stressful than being able to use the skills which they have acquired and have a high sense of self-effectiveness, which they can utilize in active combat. On the other hand, there are many triggers and stimuli in the combat environment that could lead to fear conditioning.

## Novel biological methodologies (e.g., optogenetics)

Advanced techniques that are available in animal models can enhance the time, brain-site and cell-type resolution of our understanding of gene–environment interactions relevant for PTSD susceptibility (Tye & Deisseroth, 2012). For example, optogenetic activation in mPFC of susceptible mice after chronic social defeat stress produces a rapid alleviation of social avoidance and anhedonia phenotypes seen in the socially defeated animals (Covington et al., 2010). This treatment-like response lasted for several days after the brief stimulation period. Optogenetics has also been used to demonstrate that stress-induced abnormalities in the excitability of dopamine neurons in the VTA are related to susceptibility vs. resilience to chronic social defeat stress (Chaudhury et al., 2013). Susceptibility is associated with increased firing of only those dopamine neurons that project to NAc, while those that project to mPFC show reduced firing. Furthermore, optogenetic activation of the VTA to NAc dopamine neurons is sufficient to induce a state of susceptibility, whereas optogenetic inhibition of these neurons makes susceptible mice resilient. In contrast, while optogenetic activation of the VTA to mPFC dopamine neurons was without effect, optogenetic inhibition of these neurons induced susceptibility (Chaudhury et al., 2013). This demonstrates both the need for, and power of, such specific neuronal manipulations.

## Design of an experiment

**Fig. 2:**
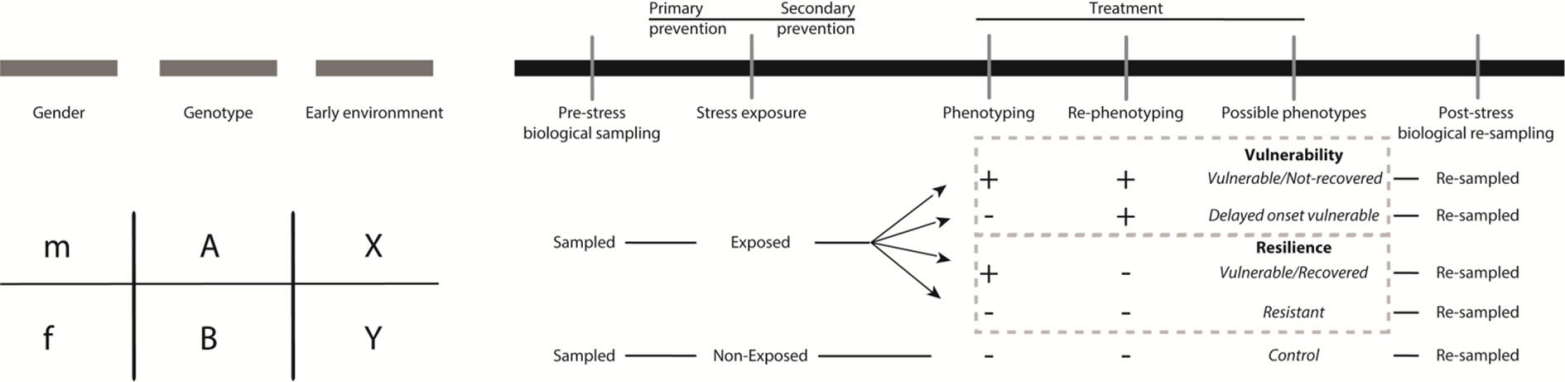
Theoretical longitudinal experimental design using an animal model of PTSD. On the left part, three predisposing factors (gender, genotype, and early environment) are depicted on a gray discontinued line which could be examined or controlled for in an animal experiment. In the right black continuous bar, the experimental design includes sampling, stress-exposure, behavioral testing and re-sampling. The time windows for primary/secondary prevention and treatment are also depicted. Pre-stress sampling is important for the discovery of a priori differences that could have predictive value on post-stress phenotypes. Yet, the possible tissue-types for sampling are limited. Stress-exposure depending on the animal model may include a single, repeated or multiple stressors. Behavioral testing should be repeated (phenotyping, re-phenotyping) to evaluate persistence of phenotypes or to detect phenotypes with delayed onset. According to phenotyping/re-phenotyping outcome (–, +) exposed animals can be classified in “Vulnerable/Not-recovered,” “Delayed onset vulnerable,” “Vulnerable/Recovered,” and “Resistant.” Often in literature the terms “Vulnerable/Not-Recovered” and “Delayed onset vulnerable” are merged into the term “Vulnerability” and “Vulnerable/Recovered” and “Resistant” are merged into “Resilience.” Phenotyping/re-phenotyping can differentiate between the overlapping groups. Post-stress re-sampling can be performed after behavioral testing with the advantage of more extensive tissue collection and the disadvantage of the numerous factors (e.g., behavioral testing) that can influence the biological material apart from stress-exposure and group differences.

This review presents and discusses recent studies using animal models that could be relevant for military PTSD. Based on these studies, we designed an experimental setup (Fig. 2) that will allow the identification of biomarkers and drug targets. Such a design includes predisposing factors (for insights on the *a priori* individual variation), biological sampling before and after stress (for identifying biological markers that have predictive value), and repeated phenotyping (for proper classification of the animals).

## Concluding Principles

We propose the following five principles for developing animal models of military PTSD:

1. The biomarker identification using exposure-based animal models will be facilitated by the use of multiple stress models that resemble different dimensions of military experience. Studying them together will permit the dissection of common neuronal and molecular networks associated with the behavioral response to traumatic events from those that might pertain to specific types of exposures or behaviors (e.g., Silva et al., 2013).
2. The duration of exposures and the frequency of heightened vigilance and extreme threat need to be better characterized in animal models of military PTSD in order to demonstrate the role of sensitization and cumulative stress exposure as critical to understanding PTSD, particularly in the combat environment.
3. Performing stress dose response experiments in large populations of outbred and inbred rodent strains can provide the link between severity of stress and PTSD.
4. Individual variability in animal work will provide insight into characteristics (e.g., genetics, developmental events) that increase susceptibility to develop PTSD after stress exposure in persons who are behaviorally no different from anyone else at baseline conditions. Focusing on discriminating biological differences between animals showing more impairment and behavioral disruption and those demonstrating less impairment, it is possible to identify biomarkers associated with vulnerability (posttrauma illness) vs. resilience (fewer and less sustained maladaptive behaviors) to similar exposures.
5. The use of genetically modified mice (or rats), viral gene transfer and optogenetic approaches to manipulate identified biomarkers, biochemical pathways, and neural circuits, provide an unprecedented advantage of highly specific and nuanced alterations of biological activity.
